# Disseminated tumor cells as selection marker and monitoring tool for secondary adjuvant treatment in early breast cancer. Descriptive results from an intervention study

**DOI:** 10.1186/1471-2407-12-616

**Published:** 2012-12-22

**Authors:** Marit Synnestvedt, Elin Borgen, Erik Wist, Gro Wiedswang, Kjetil Weyde, Terje Risberg, Christian Kersten, Ingvil Mjaaland, Lise Vindi, Cecilie Schirmer, Jahn Martin Nesland, Bjørn Naume

**Affiliations:** 1 Department of Oncology, Oslo University Hospital, Radiumhospitalet, Oslo, Norway; 2 Department of Pathology, Oslo University Hospital, Radiumhospitalet, Oslo, Norway; 3 Department of Oncology, Oslo University Hospital, Ullevål, Oslo, Norway; 4 Department of Surgery, Oslo University Hospital, Ullevål, Oslo, Norway; 5 Department of Oncology, Sykehuset Innlandet, Gjøvik, Norway; 6 Department of Oncology, University Hospital Northern Norway, Tromsø, Norway and Department of Clinical Medicine, University of Tromsø, Tromsø, Norway; 7 Department of Oncology, Sørlandet Hospital, Kristiansand, Norway; 8 Department of Oncology, Stavanger University Hospital, Stavanger, Norway; 9 Department of Oncology, Ålesund Hospital, Ålesund, Norway; 10 Department of Pathology, Oslo University Hospital, Radiumhospitalet, Oslo, Norway and Institute of Clinical Medicine, University of Oslo, Oslo, Norway; 11 Department of Oncology, Oslo University Hospital, Oslo, Norway and K.G. Jebsen Centre for Breast Cancer Research, Institute for Clinical Medicine, University of Oslo, Oslo, Norway

## Abstract

**Background:**

Presence of disseminated tumor cells (DTCs) in bone marrow (BM) after completion of systemic adjuvant treatment predicts reduced survival in breast cancer. The present study explores the use of DTCs to identify adjuvant insufficiently treated patients to be offered secondary adjuvant treatment intervention, and as a surrogate marker for therapy response.

**Methods:**

A total of 1121 patients with pN1-3 or pT1c/T2G2-3pN0-status were enrolled. All had completed primary surgery and received 6 cycles of anthracycline-containing chemotherapy. BM-aspiration was performed 8-12 weeks after chemotherapy (BM1), followed by a second BM-aspiration 6 months later (BM2). DTC-status was determined by morphological evaluation of immunocytochemically detected cytokeratin-positive cells. If DTCs were present at BM2, docetaxel (100 mg/m^2^, 3qw, 6 courses) was administered, followed by DTC-analysis 1 month (BM3) and 13 months (BM4) after the last docetaxel infusion.

**Results:**

Clinical follow-up (FU) is still ongoing. Here, the descriptive data from the study are presented. Of 1085 patients with a reported DTC result at both BM1 and BM2, 94 patients (8.7%) were BM1 positive and 83 (7.6%) were BM2 positive. The concordance between BM1 and BM2 was 86.5%. Both at BM1 and BM2 DTC-status was significantly associated with lobular carcinomas (p = 0.02 and p = 0.03, respectively; chi-square). In addition, DTC-status at BM2 was also associated with pN-status (p = 0.009) and pT-status (p = 0.03). At BM1 28.8% and 12.8% of the DTC-positive patients had ≥2 DTCs and ≥3 DTCs, respectively. At BM2, the corresponding frequencies were 47.0% and 25.3%. Of 72 docetaxel-treated patients analyzed at BM3 and/or BM4, only 15 (20.8%) had persistent DTCs. Of 17 patients with ≥3 DTCs before docetaxel treatment, 12 patients turned negative after treatment (70.6%). The change to DTC-negativity was associated with the presence of ductal carcinoma (p = 0.009).

**Conclusions:**

After docetaxel treatment, the majority of patients experienced disappearance of DTCs. As this is not a randomized trial, the results can be due to effects of adjuvant (docetaxel/endocrine/trastuzumab) treatment and/or limitations of the methodology. The clinical significance of these results awaits mature FU data, but indicates a possibility for clinical use of DTC-status as a residual disease-monitoring tool and as a surrogate marker of treatment response.

**Trial registration:**

Clin Trials Gov NCT00248703

## Background

The introduction of systemic adjuvant therapy has improved the survival of patients with early breast cancer. However, there is a lack of established tools to measure the direct effect of a given systemic treatment on minimal residual disease/micrometastases after primary surgery.

Techniques for identification and characterization of disseminated tumor cells may open possibilities for prediction of treatment response and tailored treatment decisions. Immunocytochemical detection (ICC) of disseminated tumor cells (DTCs) in the bone marrow (BM) and further analyses of these cells have been introduced as means to meet these needs [1-9]. Moreover, presence of DTCs during relapse-free follow-up (+/- tamoxifen) has been found to be a strong predictor of systemic relapse and breast cancer death [10-12]. Similar results were also reported in two smaller studies analyzing DTC status in very high-risk breast cancer patients early after completion of chemotherapy [1,13]. The presence of DTCs after chemotherapy clearly indicates a rationale for testing of alternative (secondary) treatment approaches. Detection of DTCs following treatment intervention should also be further tested for potential value as a surrogate marker for future relapse/treatment effect. During the last decade, docetaxel has been established as a highly active treatment against breast cancer. Response rates of 40-50% have regularly been reported in the metastatic setting [14,15]. Prior to the initiation of this study, results from several trials indicated that use of docetaxel in addition to anthracycline could improve the outcome of patients compared to non-taxane regimens [16-18].

In the current study*,* DTC-status was monitored after completion of anthracycline-containing adjuvant chemotherapy and used to identify high-risk patients as candidates for secondary treatment with docetaxel. The BM-status was analyzed 2-3 months (BM1) and 8-9 months after chemotherapy (BM2). To reduce inclusion of patients who might be in the process of gradual eradication of DTCs caused by an effective standard treatment, BM2 was chosen as the time point for decision about docetaxel treatment. There was also some support from previous studies to assess BM-status between 6 and 12 months after chemotherapy [13,19]. DTC-status was also explored as a surrogate marker for response by monitoring changes in DTCs after the docetaxel treatment. The clinical follow-up is still ongoing. Here, we present the descriptive data from the study.

## Methods

### Patients

A total of 1121 patients with node positive or high-risk node negative disease (pT1c/T2G2-3pN0) were enrolled in the period from October 2003 to May 2008 at 7 hospitals in Norway. All patients had completed primary surgery and 6 cycles of adjuvant anthracycline-containing chemotherapy (FEC: 5-FU 600 mg/m^2^, epirubicin 60-100 mg/m^2^ and cyclophosphamide 600 mg/m^2^ 3qw). Patients between 18-70 years with no earlier or concomitant carcinoma (other than breast carcinoma), except for basal cell carcinoma of the skin and in situ cervix cancer, were eligible if they had completed staging analysis including chest X-ray, bone scintigraphy or MRI, liver ultrasound or liver CT scan, without presence of metastases. The study was approved by the Regional Ethical Committee (reference number S-03032). Written consent was obtained from all patients. The study is registered in Clin Trials Gov (registration number NCT00248703).

Patients with estrogen receptor (ER) and/or progesteron receptor (PR) positive tumors received endocrine treatment according to standard recommendations at the time of the study (tamoxifen for 5 years; tamoxifen for 2-3 years followed by aromatase inhibitor for 2-3 years for postmenopausal patients from 2005). From June 2005, patients with HER2-positive tumors received trastuzumab every 3rd week for 1 year. This treatment was started after completion of radiotherapy. No patients received bisphosphonates as adjuvant treatment.

### Study design

**Figure 1:**
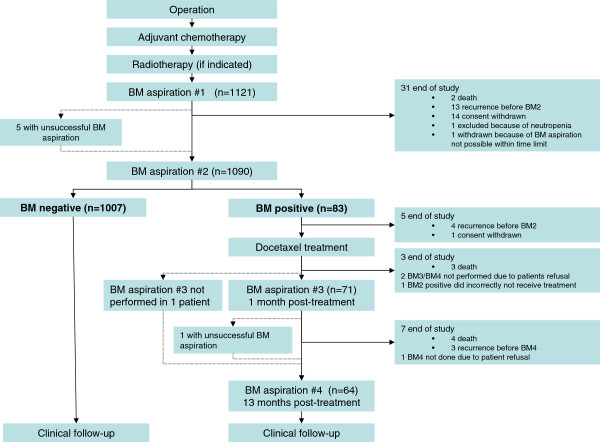
Study overview and enrollment.

The first BM-aspiration was performed at the end of radiation therapy or 8-12 weeks after standard adjuvant chemotherapy (BM1). A second BM-aspiration was performed 6 months later (BM2). Bone marrow was aspirated from posterior iliac crest bilaterally (5 ml from each site) in local anaesthesia. The processing of BM and method for DTC-analysis were performed as previously described [20]. If DTC-positive at BM2, the patient received docetaxel (100 mg/m^2^ i.v., 3qw, 6 courses). Docetaxel-treated patients were reexamined at the inclusion hospital with new BM-analysis at approximately 1 month (BM3) and 13 months (BM4) after the last docetaxel infusion. Irrespective of the DTC-status at BM2, all patients are controlled at 6-12 months interval. Study overview is shown in Figure 1. Statistical analyses that include clinical outcome are first allowed after completion of follow-up and database lock. The follow-up is still ongoing.

### Preparation of bone marrow mononuclear cell samples and detection of DTC

The BM was processed as described previously [20]. The BM-aspirates were pooled and separated by density centrifugation, mononuclear cells were collected and resuspended to 1×10^6^ cells/ml. Cytospins were prepared by centrifugation of the BM mononuclear cells (MNC) down to poly-L-lysine-coated glass slides (5 × 10^5^ MNC/slide), air-dried over night and stored at -80°C until immunostaining.

Prior to immunostaining, the cytospins were fixed for 10 min in acetone. Briefly, four slides (totally 2 × 10^6^ BM MNC) were incubated with the anti-cytokeratin monoclonal antibodies (mAbs) AE1 (Millipore, prod.no. MAB1612) and AE3 (Millipore, prod.no. MAB1611). In parallel, the same numbers of slides (2 × 10^6^ BM MNC) were incubated with the same concentration of a negative control mAb of same isotype (IgG_1_; MOPC21, Sigma, prod. no. M9269). The visualization step included incubation with polyclonal rabbit anti-mouse immunoglobulins followed by preformed complexes of alkaline phosphatase/monoclonal mouse anti-alkaline phosphatase (APAAP detection system, Dako). The color reaction was developed by incubation with New Fuchsin solution containing naphtol-AS-BI phosphate and levamisole, and the slides were counterstained with hematoxylin for 30 seconds to visualize nuclear morphology.

The slides were screened by an automated microscopy screening device (Ariol SL50, Applied Imaging), or screened manually in light microscopy. Candidate immunopositive cells selected by the automated screening were reviewed by a pathologist (E.B.). Immunopositive cells with morphology compatible with tumor cells and/or lacking hematopoietic characteristics were recorded as positive, according to the recommended guidelines [10,12,21,22]. If morphologically similar cells were detected both in the specific test and in the corresponding negative control, the result was regarded as DTC-negative. In a different cohort [20], we have tested the prognostic significance of these “double positive cases”, and no difference in clinical outcome compared to DTC-negative cases was observed (unpublished observations). In case of indeterminate cell morphology a second pathologist was consulted and consensus obtained. In doubtful cases, 16 additional cytospins were analyzed by the same ICC method (8 slides stained with AE1/AE3 mAbs and 8 slides stained with the MOPC21 control mAb).

### Analysis of primary tumor and axillary lymph node

Analysis of the primary tumors and the sentinel nodes/axillary lymph nodes were processed on a routine diagnostic basis. Histological tumor type, tumor size, and nodal involvement were analyzed and the disease was staged according to the tumor-node-metastasis (TNM) system (Union Internationale Contre le Cancer 1997). Tumor grading was performed according to Elston and Ellis [23]. The ER, PR and HER2 analyses were performed at the participating hospitals as part of the primary diagnostics. Immunohistochemical analyses for ER and PR receptors in primary tumors were performed according to the standard procedure in Norway at the time of the study and considered positive if > 10% of tumor cells stained positive with anti-ER- and/or anti-PR antibodies. The HER2 analysis was introduced as part of clinical routine from about June 2005, in parallel with the inclusion of trastuzumab into the adjuvant treatment guidelines for HER2-positive patients. Accessible HER2 results of the patients enrolled from this time period on have been obtained.

### Statistics

The SPSS software (version 18) was used for all statistical analyses. Chi-square-based tests were used for calculation of p-values for the association between baseline characteristics and bone marrow results. For all statistical calculations the Exact Sig. (2-sided) were used as follows: Fisher Exact test for variables with two categories; Linear-by-Linear Association for variables with more than two categories.

## Results

### Characteristics of the study and patients

The study overview and patient enrollment are illustrated in Figure 1 and the clinico-pathological features of the patients are shown in Table 1. The median age at inclusion was 48 years (range 23-69 years). Most of these patients had pT1c and pT2 tumors, (46.7% and 41.9% respectively) and the majority was grade 2 or 3 tumors (53.9% and 37.3% respectively). Infiltrating ductal carcinoma constituted 82.4% of the cases, while 9.8% were lobular carcinomas. Estrogen receptors were expressed in 75.2% of the cases, and 17.4% of the patients analysed for HER2 (from June 2005) were positive. Lymph node status was negative in 43.3% of the patients. Of those with presence of axillary metastases 2/3 were pN1.

**Table 1:** **Clinico-pathological data of the patients and BM-status at BM1 and BM2**^**a**^

	**All patients Number (%)**^**b**^		**BM1 neg Number (%)**^**c**^		**BM1 pos Number (%)**^**c**^		***P *value**^**d**^	**BM2 neg Number (%)**^**c**^		**BM2 pos Number (%)**^**c**^		***P *value**^**d**^
Number:	n = 1090		n = 991		n = 94			n = 1002		n = 83		
Age at inclusion (median): 48 (range 23-69)												
Menopausal status:												
Pre	592	(55.7)	540	(91.5)	50	(8.5)	0.19	547	(92.7)	43	(7.3)	0.60
Post	325	(30.6)	287	(88.9)	36	(11.1)		296	(91.6)	27	(8.4)	
Unknown	146	(13.7)	138	(95.2)	7	(4.8)		134		11	(7.6)	
Missing	27		26		1			25	(92.4)	2		
Primary breast cancer surgery:							1.00					0.29
Mastectomy	424	(39.4)	384	(91.2)	37	(8.8)		384	(91.2)	37	(8.8)	
Lumpectomy	653	(60.6)	594	(91.2)	57	(8.8)		60612	(93.1)	45	(6.9)	
Missing	13		13							1		
pT-status:							0.06					0.03
pT 1a + b	67	(6.3)	55	(82.1)	12	(17.9)		65	(97.0)	2	(3.0)	
pT1c	500	(46.7)	451	(90.7)	46	(9.3)		464	(93.4)	33	(6.6)	
pT2	449	(41.9)	418	(93.3)	30	(6.7)		407	(90.8)	41	(9.2)	
pT3	49	(4.6)	44	(91.7)	4	(8.3)		43	(89.6)	5	(10.4)	
pT4	6	(0.6)	6	(100)	0	(0)		5	(83.3)	1	(16.7)	
Missing	19		17		2			18		1		
pN-status:							0.59					0.009
pN0	462	(43.3)	424	(92.2)	36	(7.8)		428	(93.0)	32	(7.0)	
pN1	455	(42.6)	408	(89.9)	44	(9.7)		424	(93.8)	28	(6.2)	
pN2	99	(9.3)	93	(93.9)	6	(6.1)		86	(86.9)	13	(13.1)	
pN3	52	(4.9)	46	(88.5)	6	(11.5)		43	(82.7)	9	(17.3)	
Missing	22		20		2			21		1		
Histology:							0.02^e^					0.03^e^
IDC	888	(82.4)	815	(92.3)	68	(7.7)		821	(93.0)	62	(7.0)	
ILC	106	(9.8)	90	(84.9)	16	(15.1)		92	(86.8)	14	(13.2)	
Others	84	(7.8)	74	(88.1)	10	(11.9)		79	(94.0)	5	(6.0)	
Missing	12		12					10		2		
Histological grade:							0.06					0.92
Grade 1	83	(7.7)	75	(90.4)	8	(9.6)		80	(96.4)	3	(3.6)	
Grade 2	583	(53.9)	520	(89.8)	59	(10.2)		527	(91.0)	52	(9.0)	
Grade 3	404	(37.3)	378	(93.8)	25	(6.2)		376	(93.3)	27	(6.7)	
Unclassified	12	(1.2)	117	(91.7)	1	(8.3)		12	(100)	0	(0)	
Missing	8				1			7		1		
ER-status:							0.20					0.69
Pos	814	(75.2)	737	(91.0)	73	(9.0)		749	(92.5)	61	(7.5)	
Neg	268	(24.8)	250	(93.6)	17	(6.4)		245	(91.8)	22	(8.2)	
Missing	8		4		4			8				
PR-status:												
Pos	701	(64.9)	636	(91.1)	62	(8.9)	0.91	648	(92.8)	50	(7.2)	0.34
Neg	369	(34.2)	337	(91.6)	310	(8.4)		3359	(91.0)	33	(9.0)	
Unclassified	10	(1.0)	9	(100)	1	(0)		10	(100)	0	(0)	
Missing	10		9									
Endocrine therapy:							0.52					0.41
Yes	810	(76.2)	733	(90.8)	74	(9.2)		750	(92.9)	57	(7.1)	
No	253	(23.8)	233	(92.5)	19	(7.5)		230	(91.3)	22	(8.7)	
Missing	27		25		1			22		4		
HER2-status^f^:							0.46					0.84
Pos	117	(17.4)	109	(94.0)	7	(6.0)		108	(93.1)	8	(6.9)	
Neg	557	(82.6)	507	(91.4)	48	(8.6)		519	(93.5)	36	(6.5)	
Missing	51		48		2			49				
BM1:												
Positive								79	(84.0)	15	(16.0)	0.004
Negative								923	(93.1)	68	(6.9)	

^a^All patients with a BM2 result are presented (column all patients). For the data according to BM1 and BM2 status, patients with a reported result on both analyses, are presented, n = 1085) (also see Figure 1).

^b^Valid percent.

^c^The percentages for the BM1 and BM2 analysis, in relation to the clinico-pathological variables.

^d^Fisher Exact test for variables with two categories; Linear-by-Linear Association for variables with more than two categories.

“Unknown”, “Others”, Missing and “Unclassified” are not included in the statistical analysis.

^e^Comparison of infiltrating ductal carcinoma (IDC) and infiltrating lobular carcinoma (ILC).

^f^Patients enrolled from June 2005 (n = 725); HER2 testing was not performed routinely before this time point.

### DTC detection at BM1 and BM2

Out of 1085 patients with a reported DTC result for both BM1 and BM2, 94 (8.7%) and 83 patients (7.6%) were BM1 and BM2 positive, respectively (Table 1). The concordance between BM1 and BM2 was 86.5%. Among the BM1 positive patients, only 15 (16.0%) were BM2 positive, a result that may be affected by the recent administration of the standard adjuvant chemotherapy. Moreover, a change from BM1 positive to BM2 negative DTC-status was observed in 82.4% (61/74) of the endocrine treated patients, in 87.5% (7/8) of the trastuzumab treated patients (4 of the patients were treated with both endocrine therapy and trastuzumab) and in 85.7% (6/7) of the patients that did not receive endocrine treatment or trastuzumab. Presence of DTCs at BM1 was significantly associated with lobular carcinoma (p = 0.02) and BM2-status (p = 0.004), and borderline significance was observed for pT-status (p = 0.06) and histological grade (p = 0.06). At BM2, DTC-status was associated with pN-status (p = 0.009), pT-status (p = 0.03) and lobular carcinoma (p = 0.03). At BM1 28.8% of the DTC-positive patients had ≥2 DTCs, and 12.8% harboured ≥3 DTCs. At BM2, ≥2 DTCs were detected in almost half (47.0%) of the patients, whereas 25.3% had ≥3 DTCs (Table 2).

**Table 2:** Number of DTCs detected in BM1 and BM2 positive cases

**Number of DTCs**	**BM1 Number (%)**	**BM2 Number (%)**
1	67 (71.3)	44 (53.0)
2	15 (16.0)	18 (21.7)
3-9	9 (9.6)	12 (14.5)
≥10	3 (3.2)	9 (10.8)

### DTC-monitoring and tumor characteristics in docetaxel treated patients

Patients with BM2 positivity received docetaxel treatment. BM-aspiration post-treatment was performed if at least 4 cycles with docetaxel were administered. A presentation of the absolute numbers of DTCs (categorized as 0, 1, 2, 3-9 or ≥10 DTCs) at BM2, BM3 and BM4 is shown in Additional file 1: Figure S1 and Additional file 2: Table S1. At BM3 DTC-status turned negative in 59 of 71 cases (83.0%), and 53 of 64 were negative at BM4 (82.8%) (Table 3). In 19 of the patients BM-aspiration was not performed at BM3 and/or BM4, as explained in Figure 1. Of 72 patients categorized according to the last (of BM3 or BM4) performed BM-aspiration, only 15 (20.8%) had persistent DTCs after docetaxel treatment (Table 3). Of 17 patients with ≥3 DTCs before docetaxel treatment, only 5 patients were positive after treatment (29.4%).

**Table 3:** **Number of DTCs detected before (BM2) and after docetaxel treatment (BM3/BM4) in BM2-positive patients**^**a**^

**Number of DTCs**	**BM2 Number (%)**	**BM3 Number (%)**	**BM4 Number (%)**	**Last post-treatment BM result Number (%)**
0		59 (83.1)	53^b^ (82.8)	57 (79.2)
1	39 (54.1)	7 (9.9)	9 (14.1)	9 (12.5)
2	16 (22.2)	1 (1.4)	1 (1.6)	2 (2.8)
≥3	17 (23.6)	4 (5.6)	1 (1.6)	4 (5.6)
Not performed		1	8	

^a^Only patients with DTC results at BM3 and/or BM4 are included (see Figure 1).

^b^Three samples classified as negative with morphologically similar cells detected in the corresponding negative control.

Subgroup analyses of patients with persistent DTCs after treatment compared to those with negative DTC-status after treatment are shown in Table 4. The change to negative DTC-status was significantly associated with ductal carcinoma histology (p = 0.009). For the other clinico-pathological parameters there were no significant associations. Furthermore, as shown in Additional file 3: Table S2, patients with ≥3 DTCs before treatment (i.e. at BM2) who turned DTC-negative after treatment, had similar characteristics as all the patients achieving negative DTC-status.

**Table 4:** **Analyses of clinico-pathological data and DTC-status after treatment**^**a**^

**Histopathology**	**All patients Number (%)**^**b**^		**Persistent DTC after treatment Number (%)**^**c**^		**Negative for DTC after treatment Number (%)**^**c**^		***P *value**^**d**^
Number:	n=72		n=15		n=57		
pT-status:							
pT1a +b	2	(2.8)	0	(0)	2	(100)	
pT1c	29	(40.3)	6	(20.7)	23	(79.3)	0.80
≥pT2	41	(56.9)	9	(22.0)	32	(78.0)	
Histology:							
IDC	54	(76.1)	8	(14.8)	46	(85.2)	
ILC	14	(19.7)	7	(50.0)	7	(50.0)	0.009^e^
Others	3	(4.2)	0	(0)	3	(100)	
Missing	1		0		1		
ER-status:							
Pos^f^	54	(75.0)	13	(24.1)	41	(75.9)	0.33
Neg	18	(25.0)	2	(11.1)	16	(88.9)	
PR-status:							
Pos^f^	46	(63.9)	8	(17.4)	38	(82.6)	0.38
Neg	26	(36.1)	7	(26.9)	19	(73.1)	
BM1:							
Pos	13	(18.1)	5	(38.5)	8	(61.5)	0.11
Neg	59	(81.9)	8	(14.5)	47	(85.5)	
pN-status:							
pN0	28	(39.4)	4	(14.3)	24	(85.7)	0.37
pN+	43	(60.6)	11	(25.6)	32	(74.4)	
Missing	1		0		1		
Histological grade:							
Grade1-2	51	(71.8)	12	(23.5)	39	(76.5)	0.53
Grade3	20	(28.2)	3	(15.0)	17	(85.0)	
Missing	1		0		1		
HER2-status^g^:							
Pos	6	(15.8)	0	(0)	6	(100)	0.57
Neg	32	(84.2)	7	(21.9)	25	(78.1)	
Missing	1				1		

^a^Only patients with DTC results at BM3 and/or BM4 are included (see Figure 1).

^b^Valid percent.

^c^The percentages in relation to the clinico-pathological variables.

^d^Fisher exact.

^e^Comparison of infiltrating ductal carcinoma (IDC) and infiltrating lobular carcinoma (ILC).

^f^Patients with positive ER and/or PR-status received endocrine treatment.

^g^Patients enrolled from June 2005 (n = 39). Patients with positive HER2-status received trastuzumab.

For the patients with DTC presence at BM1 and/or BM2, the DTC results at all performed time points, together with the endocrine and trastuzumab treatment status, are listed in Additional file 4: Table S3.

## Discussion

The present study is, to our knowledge, the first reported study to use DTC-status to select for and monitor secondary adjuvant chemotherapy intervention in breast cancer. The identification of high-risk patients for future relapse, at a time point where otherwise no additional prognostic information can be achieved from standard histopathological/clinical assessment, is attractive. This opens for testing of alternative treatment strategies in a “window of opportunity” for potential eradication of minimal residual disease. The results show that persistent DTCs 8-9 months after 6 courses of FEC chemotherapy are changed to DTC-negativity in 79.2% of the cases following secondary treatment with docetaxel. This indicates a potential for docetaxel to eradicate minimal residual disease burden in high-risk patients. It cannot be excluded that presence of 1 DTC can be followed, by chance, by a negative result in the next test (Poisson distribution and/or methodological limitations). However, persistent negativity at two time points, and especially the fact that ¾ of the patients with ≥3 DTCs at BM2 turned negative at BM3/4, suggest a change in the tumor cell load after the intervention. The recent meta-analysis of 14 randomized clinical trials by Jean-Philippe Jacquin et al [24], support a clear additional effect of docetaxel-containing adjuvant chemotherapy to a non-taxane-containing regimen in patients with early stage breast cancer (HR 0.84 (95% CI 0.78-0.89; P < 0.001) for DFS and 0.86 (0.78-0.94; P < 0.001) for OS). The benefit is consistent across all patient subgroups, although proliferation status was not analyzed. These results may support an association between the docetaxel secondary adjuvant treatment and the reduction in DTC-positivity in our study.

The subgroup analysis shown in Table 4 reveals a significant difference in the fate of DTCs after docetaxel treatment according to histological tumor subtype. Half of the patients with lobular carcinoma had persistent DTCs, as compared to 15% of the ductal carcinoma patients. This observation is in line with a reported relative chemotherapy resistance for lobular carcinoma [25-27], and adds further support to the possibility of docetaxel-induced changes in the observed DTC-status. Furthermore, a higher fraction of patients with DTC-positive status at BM1 seemed to have persistence of DTCs after the treatment, although statistical significance was not reached (p = 0.11). It may be speculated whether a proportion of these patients have a more resistant disease (i.e. less fluctuations of DTCs despite chemotherapy). It is known from several studies that patients with primary resistance to first line chemotherapy also have a higher risk of not responding to second line treatment [28,29]. The clinical outcome of the patients included in the present study needs to be awaited, before further interpretation of the results.

The analysis of BM2 showed that DTC-status was associated with pN-status and pT-status (Table 1). The same was reported in a different study from our group, analyzing DTC-status 3 years after diagnosis [12]. In this previous study it was also observed that DTC-positivity was positively associated with lobular carcinoma, which can be explained by a relative resistance also to the anthracycline-containing chemotherapy [25,30]. Our study supports the selection of higher stage patients into future DTC intervention trials (Table 1), in order to select those with both the traditionally highest risk of relapse and the highest frequency of DTC-positivity. A consideration of the histological type may also be of importance, for selection to the proper type of systemic treatment to the right tumor subtype.

The testing of novel therapeutical principles or drugs is highly resource demanding. In addition, the effect of a new adjuvant treatment can only be evaluated when a relapse occurs, often several years later. The need for surrogate/intermediate markers to predict and monitor the therapeutic effect is obvious, but needs to be thoroughly validated. The present study is an initial step to explore the possibility to use DTCs in BM as a monitoring tool. Other possible approaches could be monitoring of circulating tumor cells (CTCs) or, as very recently reported, analysis of circulating tumor DNA in plasma/serum [31]. Detection of CTCs was not a part of the current study, because no standardized CTC-method was available at the time of study start. The performed repeated BM-analyses, however, were feasible and acceptable for the large majority of the patients.

The observed frequency of DTCs in the BM was markedly lower than what was expected prior to the study. This might be due to the assay sensitivity, but may have several additional explanations. The previously reported studies of DTCs have mostly been performed on BM-aspirates at the time of primary surgery. The subsequent administration of adjuvant chemotherapy might give a reduction in DTC-positivity. Furthermore, recent studies, using more standardized criteria, generally have shown lower DTC-positivity rates [12,20,22,32,33] than those reported in older studies. Additionally, there has been a stage migration after introduction of organized mammography screening (which is established in Norway), which probably results in less patients with micrometastatic disease. In our study, all the patients were screened for metastases before inclusion, which also might have affected the frequency of DTC presence. Finally, we used a conservative approach for inclusion of patients to docetaxel treatment in the current study. Doubtful cases were concluded as DTC-negative. It is possible to increase the sensitivity by analyzing larger number of cells (higher BM-volume). However, the clinical significance of DTC-status at primary surgery was not increased by analyzing more cells in our previous study [34]. Use of larger volumes of BM, or larger numbers of BM MNC, might require additional characterization of the detected DTCs, in order to identify markers of DTC aggressiveness and to secure both sensitivity and specificity. Accordingly, available FISH, CGH and multi-marker analyses may improve the utility of DTCs as a surrogate marker for response [33,35-39]. Characterization of the DTCs also opens for studies of tumor dormancy, EMT, stemness and/or identification of treatment targets.

The present study does not allow a direct interpretation of the effect of docetaxel on DTC-status. Although a randomized approach would have been the optimal design for this purpose, we chose the current design to explore the clinical potential for DTC-directed intervention. A randomized trial would raise several concerns, if performed un-blinded to the DTC-status. To inform the patients about a DTC-positivity without intervention (in one arm) was considered ethically difficult. A blinded study (blinded randomization of both DTC-negative and DTC-positive patients to no additional versus docetaxel treatment) was found to be premature without supporting data and would have needed a very large and expensive study. Recently a randomized trial was reported for DTC positive early breast cancer patients at diagnosis, where patients received chemotherapy +/- zoledronic acid. The results showed improved elimination of DTCs in patients treated with zoledronic acid [40]. In another study in locally advanced breast cancer, DTC status was also affected by the administration of zoledronic acid [41]. Although clinical outcome results have not yet been reported, these data support the potential use of DTCs as a monitoring tool. In our study, comparison to clinical end points has to await completion of the follow-up.

We chose 8-9 months after the standard adjuvant chemotherapy as time point for the DTC-analysis decisive for secondary adjuvant treatment. This was partially based on the results of the SBG study [13], where a positive DTC-status 6 months after chemotherapy identified patients with very poor prognosis. Furthermore, Slade et al performed repetitive BM-analyses at follow-up and found that the frequency of DTC-positive events was highest at 12 months after surgery [19]. Considering the increasing support for a detrimental outcome of patients with a positive DTC-status at later time points during follow-up [11], it might be an even more optimal approach to perform serial BM-aspirations during the first follow-up years, and to test secondary intervention whenever a DTC-positive status appear. This might be a reasonable consideration for future studies.

## Conclusions

DTC-analysis can be a useful tool for identifying patients who do not respond to a chosen standard adjuvant therapy and accordingly should be tested for benefit of additional secondary adjuvant therapy. Elimination of DTCs after docetaxel treatment was observed in the majority of the patients. Although the clinical significance of these results awaits mature follow-up data, the current study presents a novel potential approach for optimized adjuvant treatment of breast cancer, supporting further exploration of this intervention principle.

## Abbreviations

DTC: Disseminated tumor cell; BM: Bone marrow; pN1-3 and pT1c/T2G2-3pN0: Standard tumor-node-metastasis (TMN) classification according to AJCC/UICC 2002; 3qw: Every third week; FU: Follow-up; pN-status: Histopathological lymph node status; pT-status: Histopathological primary tumor size status; ER: Estrogen receptor(s); PR: Progesterone receptor(s); HER2-status: Human epidermal growth factor receptor 2; IDC: Infiltrating ductal carcinoma; ILC: Infiltrating lobular carcinoma; ICC: Immunocytochemistry; FEC: Fluorouracil epirubicine cyclophosphamide; MNC: Mononuclear cell; mAb: Mononuclear antibody; APAAP: Alkaline phosphatase/monoclonal mouse anti-alkaline phosphatase; TNM: Tumor-node-metastasis (staging system); pN1: Metastasis to 1-3 axillary lymph nodes; HR: Hazard ratio; CI: Confidence interval; DFS: Disease free survival; OS: Overall survival; FISH: Fluorescence in situ hybridization; CGH: Comparative genomic hybridization; multi-marker IF: Multi-marker immunoflourecence; EMT: Epithelial-mesenchymal transition; SBG: Scandinavian Breast Group.

## Competing interests

The authors declare that they have no competing interests.

## Authors' contributions

BN was head of study. MS, BN and EB drafted the manuscript. MS and BN performed the data analysis and carried out the statistics. BN and EW were responsible for study design. MS, EW, GW, KW, TR, CK, IM and BN were responsible for enrollment of patients. EB and JMN scored/classified the detected cells. CBS was responsible for the automated screening; EB performed the manual screening. All authors read and approved the final manuscript.

## Pre-publication history

The pre-publication history for this paper can be accessed here:

http://www.biomedcentral.com/1471-2407/12/616/prepub

## Supplementary Material

Additional file 1 **Figure S1.** Number of DTCs detected in BM2-positive, docetaxel treated patients, at BM2, BM3 and BM4. Click here for file

Additional file 2 **Table S1.** Complete DTC-status at all time points for BM2-positive patients. Click here for file

Additional file 3 **Table S2.** Analyses of clinico-pathological data and DTC-status after treatment for patients with ≥ 3 DTCs at BM2. Click here for file

Additional file 4 **Table S3.** Presentation of DTC status (at all performed time points), endocrine treatment and trastuzumab treatment status for BM1 and/or BM2 positive patients. Click here for file
