# Supplementary figures and images for: Disseminated tumor cells as selection marker and monitoring tool for secondary adjuvant treatment in early breast cancer. Descriptive results from an intervention study
Source: BMC Cancer. 2012 Dec 22;12:616. doi: 10.1186/1471-2407-12-616 (PMC3576235; doi:10.1186/1471-2407-12-616)

BM2

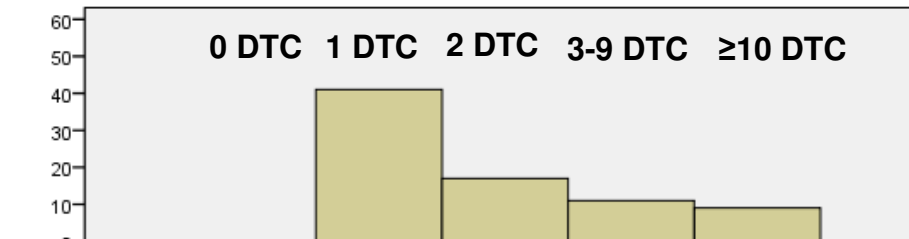

BM3

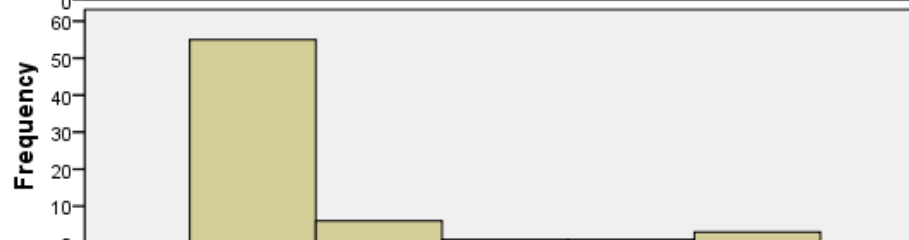

BM4

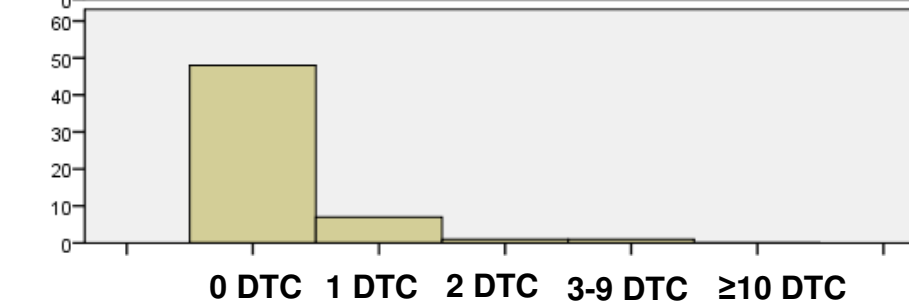

Supplement: Additional file 1 — Figure S1. Number of DTCs detected in BM2-positive, docetaxel treated patients, at BM2, BM3 and BM4. [file 1471-2407-12-616-S1.pdf]
