# Supplementary material for: Disseminated tumor cells as selection marker and monitoring tool for secondary adjuvant treatment in early breast cancer. Descriptive results from an intervention study
Source: BMC Cancer. 2012 Dec 22;12:616. doi: 10.1186/1471-2407-12-616 (PMC3576235; doi:10.1186/1471-2407-12-616)
Supplement: Additional file 2 — Table S1. Complete DTC-status at all time points for BM2-positive patients. [file 1471-2407-12-616-S2.pdf]

Supplementary table 1 Complete DTC status at all time points for BM2 positive patients

	Patient Number	BM1	BM1 #DTC	BM2 #DTC	BM3	BM3 #DTC	BM4	BM4 #DTC
1	20	neg	0 DTC	2 DTC	neg	0 DTC	neg	0 DTC
2	32	neg	0 DTC	1 DTC	neg	0 DTC	neg	0 DTC
3	33	neg	0 DTC	2 DTC	neg	0 DTC	neg	0 DTC
4	35	neg	0 DTC	2 DTC	neg	0 DTC	neg	0 DTC
5	39	neg	0 DTC	3-9 DTC	neg	0 DTC	neg	0 DTC
6	40	neg	0 DTC	1 DTC	pos	1 DTC	neg	0 DTC
7	44	neg	0 DTC	1 DTC	neg	0 DTC	neg	0 DTC
8	45	neg	0 DTC	1 DTC	pos	1 DTC	neg	0 DTC
9	47	neg	0 DTC	3-9 DTC	neg	0 DTC	Not performed	Not performed
10	65	neg	0 DTC	2 DTC	neg	0 DTC	neg	0 DTC
11	79	pos	1 DTC	1 DTC	neg	0 DTC	neg	0 DTC
12	86	neg	0 DTC	1 DTC	neg	0 DTC	pos	1 DTC
13	96	neg	0 DTC	1 DTC	neg	0 DTC	pos	1 DTC
14	97	pos	1 DTC	2 DTC	Not performed	Not performed	Not performed	Not performed
15	98	neg	0 DTC	1 DTC	neg	0 DTC	pos	1 DTC
16	115	neg	0 DTC	2 DTC	neg	0 DTC	neg	0 DTC
17	117	pos	1 DTC	1 DTC	neg	0 DTC	neg	0 DTC
18	128	neg	0 DTC	1 DTC	neg	0 DTC	neg	0 DTC
19	170	pos	1 DTC	≥10 DTC	Not performed	Not performed	Not performed	Not performed
20	171	neg	0 DTC	3-9 DTC	neg	0 DTC	neg	0 DTC
21	181	neg	0 DTC	1 DTC	neg	0 DTC	neg	0 DTC
22	185	neg	0 DTC	1 DTC	pos	1 DTC	pos	1 DTC
23	195	neg	0 DTC	1 DTC	pos	1 DTC	pos	1 DTC
24	202	neg	0 DTC	≥10 DTC	pos	≥10 DTC	Not performed	Not performed

25	205	neg	0 DTC	1 DTC	neg	0 DTC	neg	0 DTC
26	236	neg	0 DTC	1 DTC	neg	0 DTC	neg	0 DTC
27	243	neg	0 DTC	1 DTC	Not performed	Not performed	Not performed	Not performed
28	251	neg	0 DTC	2 DTC	neg	0 DTC	pos	2 DTC
29	259	neg	0 DTC	1 DTC	neg	0 DTC	neg	0 DTC
30	267	neg	0 DTC	2 DTC	neg	0 DTC	neg	0 DTC
31	277	neg	0 DTC	1 DTC	neg	0 DTC	neg	0 DTC
32	278	neg	0 DTC	1 DTC	pos	1 DTC	neg	0 DTC
33	280	neg	0 DTC	≥10 DTC	pos	≥10 DTC	Not performed	Not performed
34	311	neg	0 DTC	1 DTC	neg	0 DTC	neg	0 DTC
35	342	neg	0 DTC	2 DTC	neg	0 DTC	neg	0 DTC
36	343	neg	0 DTC	≥10 DTC	Not performed	Not performed	Not performed	Not performed
37	366	pos	1 DTC	1 DTC	pos	1 DTC	neg	0 DTC
38	383	neg	0 DTC	1 DTC	Not performed	Not performed	Not performed	Not performed
39	396	neg	0 DTC	1 DTC	neg	0 DTC	neg	0 DTC
40	440	pos	≥10 DTC	2 DTC	Not performed.	.Not performed	pos	1 DTC
41	477	neg	0 DTC	≥10 DTC	Not performed	Not performed	Not performed	Not performed
42	512	neg	0 DTC	1DTC	neg	0 DTC	neg	0 DTC
43	562	pos	≥10 DTC	≥10 DTC	pos	≥10 DTC	Not performed	Not performed
44	569	neg	0 DTC	1DTC	Not performed	Not performed	Not performed	Not performed
45	578	neg	0 DTC	3-9 DTC	neg	0 DTC	neg	0 DTC
46	587	neg	0 DTC	1 DTC	Not performed	Not performed	Not performed	Not performed
47	601	neg	0 DTC	3-9 DTC	neg	0 DTC	neg	0 DTC
48	613	neg	0 DTC	2 DTC	neg	0 DTC	neg	0 DTC
49	630	neg	0 DTC	1 DTC	pos	1 DTC	neg	0 DTC
50	648	neg	0 DTC	1 DTC	neg	0 DTC	neg	0 DTC
51	661	neg	0 DTC	1 DTC	neg	0 DTC	neg	0 DTC
52	669	neg	0 DTC	2 DTC	neg	0 DTC	neg	0 DTC

53	677	pos	3-9 DTC	3-9 DTC	neg	0 DTC	neg	0 DTC
54	695	pos	2 DTC	3-9 DTC	neg	0 DTC	pos	1 DTC
55	708	neg	0 DTC	1 DTC	neg	0 DTC	neg	0 DTC
56	740	neg	0 DTC	3-9 DTC	neg	0 DTC	neg	0 DTC
57	751	pos	1 DTC	≥10 DTC	neg	0 DTC	Not performed	Not performed
58	755	neg	0 DTC	1 DTC	neg	0 DTC	neg	0 DTC
59	766	neg	0 DTC	1 DTC	neg	0 DTC	Not performed	Not performed
60	786	neg	0 DTC	1 DTC	neg	0 DTC	neg	0 DTC
61	805	neg	0 DTC	1 DTC	neg	0 DTC	neg	0 DTC
62	813	neg	0 DTC	2 DTC	Not performed	Not performed	Not performed	Not performed
63	821	neg	0 DTC	1 DTC	neg	0 DTC	neg	0 DTC
64	839	neg	0 DTC	2 DTC	neg	0 DTC	neg	0 DTC
65	852	neg	0 DTC	2 DTC	neg	0 DTC	neg	0 DTC
66	858	neg	0 DTC	3-9 DTC	neg	0 DTC	neg	0 DTC
67	878	pos	1 DTC	1 DTC	neg	0 DTC	neg	0 DTC
68	895	neg	0 DTC	1 DTC	neg	0 DTC	neg	0 DTC
69	908	neg	0 DTC	3-9 DTC	neg	0 DTC	neg	0 DTC
70	928	neg	0 DTC	1 DTC	neg	0 DTC	neg	0 DTC
71	929	neg	0 DTC	1 DTC	neg	0 DTC	neg	0 DTC
72	964	neg	0 DTC	≥10 DTC	Not performed	Not performed	Not performed	Not performed
73	983	neg	0 DTC	1 DTC	neg	0 DTC	pos	1 DTC
74	1009	neg	0 DTC	2 DTC	neg	0 DTC	Not performed	Not performed
75	1031	neg	0 DTC	1 DTC	pos	3-9 DTC	neg	0 DTC
76	1045	pos	2 DTC	2 DTC	neg	0 DTC	neg	0 DTC
77	1049	neg	0 DTC	1 DTC	Not performed	Not performed	Not performed	Not performed
78	1054	pos	1 DTC	3-9 DTC	neg	0 DTC	neg	0 DTC
79	1061	pos	1 DTC	1 DTC	pos	2 DTC	Not performed	Not performed
80	1062	neg	0 DTC	≥10 DTC	neg	0 DTC	neg	0 DTC

81	1078	neg	0 DTC	2 DTC	neg	0 DTC	pos	1 DTC
82	1100	neg	0 DTC	1 DTC	neg	0 DTC	neg	0 DTC
83	1122	pos	2 DTC	3-9 DTC	neg	0 DTC	pos	3-9 DTC
