# Supplementary material for: Disseminated tumor cells as selection marker and monitoring tool for secondary adjuvant treatment in early breast cancer. Descriptive results from an intervention study
Source: BMC Cancer. 2012 Dec 22;12:616. doi: 10.1186/1471-2407-12-616 (PMC3576235; doi:10.1186/1471-2407-12-616)
Supplement: Additional file 3 — Table S2. Analyses of clinico-pathological data and DTC-status after treatment for patients with ≥ 3 DTCs at BM2. [file 1471-2407-12-616-S3.pdf]

Supplementary table 2 Analyses of clinico-pathological data and DTC-status after treatment for patients with 3≥DTCs at BM2

Histopathological data	3≥DTCs and positive for DTC after treatment (n=5) No (%)[a]		3≥DTCs and negative for DTC after treatment (n=12) No (%)[a]	
pT-status:				
pT1c	2	(33.3)	4	(66.7)
pT2	3	(27.3)	8	(72.7)
pN-status:				
pN0	1	(16.7)	5	(83.3)
pN1	2	(40.0)	3	(60.0)
pN2	2	(50)	2	(50.0)
pN3	0	(0)	2	(100)
pN0 vs. pNpos:				
pN0	1	(16.7)	5	(83.3)
pNpos	4	(36.4)	7	(63.6)
Histology:				
IDC	3	(23.1)	10	(76.9)
ILC	2	(50.0)	2	(50.0)
Histological grade:				
Grade 1	0	(0)	1	(100)
Grade 2	4	(40.0)	6	(60.0)
Grade 3	1	(16.7)	5	(83.3)
Grade 1&2 vs. Grade3:				
Grade1&2	4	(36.4)	7	(63.6)
Grade3	1	(16.7)	5	(83.3)
ER-status:				
Pos	5	(35.7)	9	(64.3)
Neg	0	(0)	3	(100)
PgR-status:				
Pos	3	(23.1)	10	(76.9)
Neg	2	(50.0)	2	(50.0)
BM1				
Positive	3	(50.0)	3	(50.0)
Negative	2	(18.2)	9	(81.8)
HER2-status[b]:				
Positive =0				
Negative	3	(25.0)	9	(75.0)

[a]The percentages in relation to the clinico-pathological variables
[b]Patients enrolled from June 2005 (n=12)
