# Supplementary material for: Disseminated tumor cells as selection marker and monitoring tool for secondary adjuvant treatment in early breast cancer. Descriptive results from an intervention study
Source: BMC Cancer. 2012 Dec 22;12:616. doi: 10.1186/1471-2407-12-616 (PMC3576235; doi:10.1186/1471-2407-12-616)
Supplement: Additional file 4 — Table S3. Presentation of DTC status (at all performed time points), endocrine treatment and trastuzumab treatment status for BM1 and/or BM2 positive patients. [file 1471-2407-12-616-S4.pdf]

Supplementary table 3
Presentation of DTC status (at all performed time points), endocrine treatment and trastuzumab treatment status for BM1 and/or BM2 positive patients

Patient Number	BM1	BM2	BM3	BM4	Endocrine treatment	Trastuzumab
5	Pos	Neg			No	No
7	Pos	Neg			No	No
12	Pos	Neg			Yes	No
13	Pos	Neg			No	No
16	Pos	Neg			Yes	No
17	Pos	Neg			Yes	No
26	Pos	Neg			Yes	No
42	Pos	Neg			Yes	No
55	Pos	Neg			Yes	No
69	Pos	Neg			Yes	No
76	Pos	Neg			Yes	No
80	Pos	Neg			Yes	No
81	Pos	Neg			Yes	No
122	Pos	Neg			No	No
139	Pos	Neg			Yes	No
150	Pos	Neg			Yes	No
173	Pos	Neg			Yes	No
174	Pos	Neg			Yes	No
209	Pos	Neg			No	No
210	Pos	Neg			Yes	No
238	Pos	Neg			No	No
254	Pos	Neg			No	Yes
269	Pos	Neg			Yes	No
289	Pos	Neg			Yes	No
323	Pos	Neg			Yes	No
331	Pos	Neg			Yes	No
339	Pos	Neg			Yes	Unknown
346	Pos	Neg			Yes	No
348	Pos	Neg			No	No
353	Pos	Neg			Yes	No
365	Pos	Neg			Yes	No
374	Pos	Neg			Yes	No
392	Pos	Neg			Yes	No
413	Pos	Neg			No	No
432	Pos	Neg			Yes	No
437	Pos	Neg			Yes	Unknown
450	Pos	Neg			Yes	No
454	Pos	Neg			Yes	No

468	Pos	Neg			Yes	No
486	Pos	Neg			Yes	No
492	Pos	Neg			Yes	No
571	Pos	Neg			Yes	Yes
574	Pos	Neg			Yes	No
608	Pos	Neg			Yes	No
628	Pos	Neg			Yes	No
642	Pos	Neg			Yes	No
659	Pos	Neg			.	No
667	Pos	Neg			Yes	No
680	Pos	Neg			Yes	No
685	Pos	Neg			Yes	No
686	Pos	Neg			Yes	No
709	Pos	Neg			Yes	No
714	Pos	Neg			No	Yes
732	Pos	Neg			No	No
736	Pos	Neg			Yes	No
739	Pos	Neg			Yes	No
763	Pos	Neg			Yes	No
764	Pos	Neg			Yes	No
774	Pos	Neg			Yes	Yes
809	Pos	Neg			Yes	No
816	Pos	Neg			Yes	No
820	Pos	Neg			No	No
822	Pos	Neg			Yes	Yes
867	Pos	Neg			Yes	No
872	Pos	Neg			Yes	No
886	Pos	Neg			Yes	No
914	Pos	Neg			Yes	No
926	Pos	Neg			Yes	No
946	Pos	Neg			Yes	No
950	Pos	Neg			Yes	No
971	Pos	Neg			Yes	No
976	Pos	Neg			No	Yes
1026	Pos	Neg			No	No
1033	Pos	Neg			Yes	No
1053	Pos	Neg			No	No
1063	Pos	Neg			No	No
1066	Pos	Neg			Yes	No
1087	Pos	Neg			Yes	Yes
1109	Pos	Neg			Yes	No
79	Pos	Pos	Neg	Neg	Yes	No
97	Pos	Pos	Not performed	Not performed	Yes	No

117	Pos	Pos	Neg	Neg	Yes	No
170	Pos	Pos	Not performed	Not performed	Yes	No
366	Pos	Pos	Pos	Not evaluable	No	No
440	Pos	Pos	Not performed	Pos	Yes	No
562	Pos	Pos	Pos	Not performed	No	No
677	Pos	Pos	Neg	Neg	Yes	No
695	Pos	Pos	Neg	Pos	Yes	No
878	Pos	Pos	Neg	Neg	Yes	Yes
751	Pos	Pos	Neg	Not performed	Yes	No
1045	Pos	Pos	Neg	Neg	Yes	No
1054	Pos	Pos	Neg	Neg	Yes	No
1061	Pos	Pos	Pos	Not performed	Yes	No
1122	Pos	Pos	Neg	Pos	Yes	No
20	Neg	Pos	Neg	Neg	No	No
32	Neg	Pos	Neg	Neg	Unknown	No
33	Neg	Pos	Neg	Neg	Yes	No
35	Neg	Pos	Neg	Neg	Yes	No
39	Neg	Pos	Neg	Neg	Yes	No
40	Neg	Pos	Pos	Neg	Yes	No
44	Neg	Pos	Neg	Neg	Yes	No
45	Neg	Pos	Pos	Neg	No	No
47	Neg	Pos	Neg	Not performed	No	No
65	Neg	Pos	Neg	Neg	Yes	No
86	Neg	Pos	Neg	Pos	Yes	No
96	Neg	Pos	Neg	Pos	Yes	No
98	Neg	Pos	Neg	Pos	Yes	No
115	Neg	Pos	Neg	Neg	Yes	No
128	Neg	Pos	Neg	Neg	Yes	No
171	Neg	Pos	Neg	Neg	No	No
181	Neg	Pos	Neg	Not evaluable	Yes	No
185	Neg	Pos	Pos	Pos	No	No
195	Neg	Pos	Pos	Pos	No	No
202	Neg	Pos	Pos	Not performed	Yes	No
205	Neg	Pos	Neg	Neg	No	No
236	Neg	Pos	Neg	Neg	Yes	No
243	Neg	Pos	Not performed	Not performed	Yes	No
251	Neg	Pos	Neg	Pos	Yes	No
259	Neg	Pos	Neg	Neg	No	No
267	Neg	Pos	Neg	Neg	Yes	No
277	Neg	Pos	Neg	Neg	Yes	No
278	Neg	Pos	Pos	Neg	Yes	Unknown
280	Neg	Pos	Pos	Not performed	Yes	No
311	Neg	Pos	Neg	Neg	Unknown	No

342	Neg	Pos	Neg	Neg	No	No
343	Neg	Pos	Not performed	Not performed	Unknown	No
383	Neg	Pos	Not performed	Not performed	Yes	No
396	Neg	Pos	Neg	Neg	No	Yes
477	Neg	Pos	Not performed	Not performed	No	No
512	Neg	Pos	Neg	Neg	Yes	Yes
569	Neg	Pos	Not performed	Not performed	No	Yes
578	Neg	Pos	Neg	Neg	Yes	No
587	Neg	Pos	Not performed	Not performed	No	No
601	Neg	Pos	Neg	Neg	Yes	No
613	Neg	Pos	Neg	Neg	Yes	No
630	Neg	Pos	Pos	Neg	No	No
648	Neg	Pos	Neg	Neg	No	No
661	Neg	Pos	Neg	Neg	Yes	No
669	Neg	Pos	Neg	Neg	Yes	No
708	Neg	Pos	Neg	Neg	No	No
740	Neg	Pos	Neg	Not evaluable	Yes	No
755	Neg	Pos	Neg	Neg	No	No
766	Neg	Pos	Neg	Not performed	No	Yes
786	Neg	Pos	Neg	Neg	Yes	No
805	Neg	Pos	Neg	Neg	Yes	No
813	Neg	Pos	Not performed	Not performed	Yes	Yes
821	Neg	Pos	Neg	Neg	Yes	No
839	Neg	Pos	Neg	Neg	Unknown	Yes
852	Neg	Pos	Neg	Neg	Yes	No
858	Neg	Pos	Neg	Neg	Yes	No
895	Neg	Pos	Neg	Neg	No	No
908	Neg	Pos	Neg	Neg	Yes	No
928	Neg	Pos	Neg	Neg	No	Yes
929	Neg	Pos	Neg	Neg	Yes	No
964	Neg	Pos	Not performed	Not performed	Yes	No
983	Neg	Pos	Neg	Pos	Yes	No
1009	Neg	Pos	Neg	Not performed	Yes	No
1031	Neg	Pos	Pos	Neg	Yes	No
1049	Neg	Pos	Not performed	Not performed	Yes	No
1062	Neg	Pos	Neg	Neg	Yes	No
1078	Neg	Pos	Neg	Pos	Yes	No
1100	Neg	Pos	Neg	Neg	Yes	No
